# LC method for the direct and simultaneous determination of four major furan derivatives in coffee grounds and brews

**DOI:** 10.1002/jssc.201900061

**Published:** 2019-03-18

**Authors:** Abdullatif Albouchi, Michael Murkovic

**Affiliations:** ^1^ Institute of Biochemistry Graz University of Technology Graz Austria

**Keywords:** coffee, extraction solvents, extraction techniques, furan derivatives, high‐performance liquid chromatography

## Abstract

The simultaneous quantification of two potential genotoxic hydroxymethyl furan derivatives in coffee (furfuryl alcohol and 5‐hydroxymethylfurfural) alongside their carboxylic acid derivatives (2‐furoic acid and 5‐hydroxymethyl furoic acid, respectively) was carried out. Their extraction from ground roasted coffee using sonication, simple shaking or heat‐assisted shaking lead to similar results. A minimum of 97.3% of the four furan derivatives were extracted during the first extraction cycle using water, whereas methanol showed considerably lower extraction efficiency. A simple high‐performance liquid chromatography method coupled with diode array detection was developed for the simultaneous determination and was applied to roasted coffee extracts or brews. No sample pre‐treatment except for centrifugation was needed. The diode array detector was used to assess the purity of the peaks of interest in analyzed samples against authentic standards. The linearity according to Mandel, accuracy (recovery ≥ 89.9%) and precision (inter‐ and intraday relative standard deviation ≤ 4.5%) were checked. The values for the limit of detection and quantification ranged within 0.11–0.76 and 0.35–2.55 μg/mL, respectively. Filtered and espresso brews were analyzed for the four furan derivatives where furfuryl alcohol showed double the concentration of 5‐hydroxymethylfurfural and about ten times the concentrations of 2‐furoic acid or 5‐hydroxymethyl furoic acid.

**Article Related Abbreviations**
2‐FA: 2‐furoic acid
FFA: furfuryl alcohol
HMF: 5‐hydroxymethylfurfural
HMFA: 5‐hydroxymethyl furoic acid

## 1 INTRODUCTION

Furan derivatives are a range of chemicals that share a five‐membered ring containing oxygen. This ring can carry one or more substituents affecting its physio‐chemical properties such as solubility and volatility 1. This class of compounds is receiving a lot of attention due to their pleasant contributions to the aroma, color and taste of many foods and beverages as well as their potentially harmful effects 2.

Coffee is one of these foods with a specific aroma related to furan derivatives. In green coffee, furans are not present naturally. They are formed during the roasting process where the level of these compounds correlates well with the brown color of roasting. It was found in several studies that this class of compounds comprises the major components of coffee aroma 1, 3. Of special importance are furfuryl alcohol (FFA) and 5‐hydroxymethylfurfural (HMF) which are found in substantial quantities in coffee along with their carboxylic acid derivatives; 2‐furoic acid (2‐FA) and 5‐hydroxymethyl furoic acid (HMFA), respectively. FFA alone accounts for more than 50% of all furans quantified 3 while HMF was the major volatile chemical in light roasted coffee 4.

On the other hand, several animal studies have proven that FFA and HMF can form DNA adducts through a similar conversion mechanism. Intracellular sulfotransferases (SULT) are involved in the production of reactive electrophiles from FFA and HMF (2‐sulfo‐oxymethylfuran and 5‐sulfooxymethylfurfural, respectively), which in turn react with the DNA leading to mutations 5, 6. 2‐FA and HMFA are natural metabolites of FFA and HMF, respectively 5, 7. In addition, HMFA is formed during coffee roasting 8.

This compiled information about the significant presence of furan derivatives as well as their toxicological effects gives rise to the importance of monitoring these compounds quantitatively in foods that are both rich in them and are also heavily consumed such as coffee. Currently, GC is the common method for the monitoring of furan derivatives given the volatility of some members of this class. However, furan derivatives form during heat treatment of food samples that contain their precursors, thus the application of heat on these samples during the injection into GC systems might lead to the formation of artefacts or can change the actual amounts of these derivatives 2. The use of HPLC avoids the application of such heat treatments. Another advantage to be mentioned is that HPLC is not a destructive analysis method, meaning that the analyzed samples can be retrieved after analysis for further examinations, which is not possible in GC. HPLC has been previously reported for the analysis of furan derivatives in apple cider and wine 2, 9, fermented soy sauce 10, traditional balsamic vinegars 11, fruit juices 12, sugarcane honey 13 and treacle 14, while publications concerning coffee focus on the analysis of a single furan derivative 15, 16, 17.

This manuscript describes the optimization of a simple and reliable analytical method for the extraction and simultaneous determination of FFA, 2‐FA, HMF and HMFA using an HPLC‐DAD system equipped with a C8 column with the aim to implement this method in the routine analysis for monitoring the content of these compounds in roasted ground coffee and their brews.

## 2 MATERIALS AND METHODS

### 2.1 Coffee sample

A commercially roasted and ground coffee was purchased from a local market. The product was labeled as a medium roast (Vienna roast) of 100% Arabica type coffee.

### 2.2 Chemicals

FFA (≥98 %) was obtained from Fluka Chemicals (Basel, Switzerland). 2‐FA (98%) and HMF (99%) were obtained from Aldrich Chemistry (St. Louis, MO, USA). HMFA was obtained from Matrix Scientific (Columbia, SC, USA). Methanol (HPLC grade) was obtained from Chem Lab NV (Zedelgem, Belgium). Water for HPLC was obtained from Ultra clear TWF‐UV system (Siemens water technologies, Germany). Glacial acetic acid (99–100%) was obtained from Avantor performance material (Deventer, Netherlands).

### 2.3 Assessment of different extraction techniques

Three different extraction techniques were applied to examine different extraction efficiencies. 50 mg of ground roasted coffee were extracted with 1 ml of distilled water in a 2 mL‐centrifugation tube. Sonication in a water bath for 10 min, simple shaking with a vortex mixer Genie 2 (Scientific Industries, USA) for 10 min or heat‐assisted shaking in a thermomixer for 10 min at 60°C and 1000 rpm were applied. The tubes were then centrifuged at 5°C and 14 000 rpm for 15 min before the supernatant was taken for HPLC analysis. Three replicates were prepared for each extraction method.

### 2.4 Assessment of sample clean‐up

For this experiment, 50 mg of ground coffee were extracted twice to determine whether the extraction was quantitative. 1000 μL of the solvent were added to a 2‐mL centrifugation tube containing the ground coffee. The total weight of the tube and its content was noted (w_1_). The tube was extracted by heating in a thermomixer for 10 min at 60°C with shaking at 1000 rpm. After extraction the tubes were centrifugated at 5°C and 14 000 rpm for 15 min. The supernatant was taken for analysis by HPLC and the amounts (in μg) of the four furan derivatives in the first extract (A_1_) were calculated for 1 g of solvent. The weight of the tube and its content after removal of the supernatant was also noted (w_2_). The residual solvent in the tube can be used to calculate the amount of each furan derivative remaining in the solvent adsorbed to the coffee (A_rem_) by Eq. (1). Then the extraction was repeated and the amounts (in μg) of each furan derivative in the second extract (A_2_) were determined for 1 g of solvent. Equation (2) was used to calculate the amounts of each furan derivative that was directly extracted by the second round (A_extra_). The value (P) corresponding to the percentage of the amount extracted by the second extraction round to the total extractable amount was calculated (Eq. (3)).

(1) A rem =A1×1000−w1−w21000

(2) A extra =A2−Arem

(3) P=A extra A1+A extra ×100

The clean‐up was assessed using pure water, 50% v/v methanol in water and 100% methanol. Each solvent‐experiment was performed in triplicate. The results obtained were compared statistically using Student's *t*‐test with the significance level set to α = 0.05.

### 2.5 HPLC analysis of furan derivatives

Two microliters of extracts, brews or standards were injected into an Agilent 1100 series HPLC system (Agilent Technologies, Germany) equipped with a diode array detector and a Zorbax Eclipse (XBD‐C8 4.6 × 150 mm, 5 μm, Agilent Technologies, USA) with a pre‐column of the same material. The separation was done using a gradient mobile phase of 0.1% acetic acid in water (A) and methanol (B) at 25°C. The gradient started with 100% A, at 2.5 min B was increased to 16%, between 10 and 10.5 min B was increased to 100% and held until the end of the run (15 min) with a flow rate of 0.5 mL/min. Detection was done at a wavelength of 217 nm for FFA. HMF was detected at 284 nm. 2‐FA and HMFA were detected at 250 nm.

### 2.6 Method validation

Five mixed standards containing FFA, 2‐FA, HMF and HMFA dissolved in water were prepared at five different concentrations (5, 10, 15, 20 and 25 μg/mL of each compound). These standards were used to establish the calibration curve and were also used through the validation procedure since it is not possible to prepare blank samples (roasted coffee without furanic compounds).

The linearity of the analytical method was determined by correlating the mean value (*n* = 3) of the peak area of each furan derivative in the mixed standard at each concentration (5, 10, 15, 20, and 25 μg/mL) against the corresponding concentration. The correlation coefficient of the determinations (*r* ^2^), regression data (slope and intercept), as well as the curve standard error were calculated. The linearity was checked with Mandel's test which evaluates the best fit for the data at hand among zero and first order regressions.

The precision of the analytical method was determined by calculating the relative standard deviation of the concentrations determined for each furan derivative at each concentration level (5, 10, 15, 20, and 25 μg/mL). For intra‐day precision, the determined concentrations in three replicates performed at the same day were used. For inter‐day precision, the determined concentrations in three replicates performed at three consecutive days were used.

The recovery of the analytical method was conducted by spiking 50 mg of the commercial roasted coffee sample with 10, 20, 30, 40, or 50 μL of furan derivatives stock solution (500 μg/mL of each of the four compounds) and then extracting with 1 mL of water using heat‐assisted shaking in a thermomixer (Eppendorf, Germany) for 10 min at 60°C and 1000 rpm. The samples were then centrifuged (Eppendorf 5804 R centrifuge, Germany) at 5°C and 14 000 rpm for 15 min before the supernatant was taken for HPLC analysis. Duplicates were prepared for each spiking level. A regression line was drawn by plotting the obtained areas against the expected theoretical concentrations of the furan derivatives after spiking. The combined multi‐level recovery was calculated as the ratio of the slope of the spiked samples regression line to the slope calibration curve. As the recovery approaches 100%, the slopes of the two lines should be identical and the ratio becomes 1.

The LOD and LOQ of the four furan derivatives were determined from signals obtained from standard solutions of known concentrations and those obtained from blank injections. The signal to noise ratio (S/N) was calculated by comparing the height of peaks from a standard injection of known concentration and the noise that was determined as the difference between the maximum and minimum noise responses from a blank solvent injection. The choice of pure water as a blank solvent was made because water is the most favorable solvent used to extract as well as to prepare standard solutions of the four furan derivatives in question. The noise was determined in a time range equal to five times the width of the standard peak at half height, this range is distributed equally around the retention time of the standard peak. LOD was determined as (3 × S/N) while LOQ was determined as (10 × S/N) 18.

### 2.7 Coffee brewing methods

The cupping ratio used in the preparation of the brews is 7 g of ground coffee per 100 mL of water as suggested by the ISO 6668: 2008 for sensory analysis of coffee brews 19.

For the preparation of filter brewed coffee, 35 g of the ground roasted coffee were weighed into a commercial filter coffee maker. 500 mL of deionized water was used for brewing.

For the preparation of espresso brewed coffee, 14 g of the ground roasted coffee were put in the metal sieve of the espresso maker. 200 mL of deionized water were used for brewing.

Two samples (1 mL each) were taken from each brew and centrifuged for 20 min at 15°C at 14 000 rpm. The supernatants were analyzed by HPLC.

## 3 RESULTS AND DISCUSSION

### 3.1 Development of the analytical method

**Figure 1:**
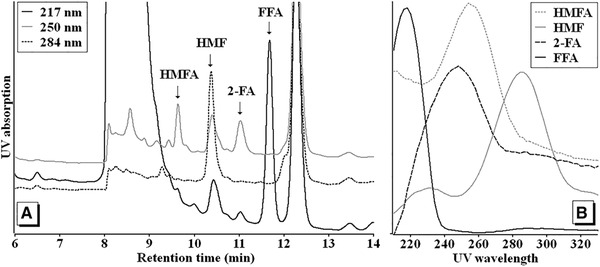
(A) HPLC chromatogram of the brew of a commercial 100% Arabica coffee indicating the peaks of FFA, HMF, 2‐FA and HMFA. (B) The baseline corrected UV absorption spectrum at the peak maximum of the four furan derivatives

A comprehensive reversed phase liquid chromatography method development was established by manipulating multiple parameters affecting the chromatographic performance. Among the four furan derivatives of interest, it was found in our experiments that the retention time of FFA was the least affected by the pH of the mobile phase. Therefore, different chromatographic columns (C18, C8, PFP, HILIC and ion exchange) were tested to achieve an optimal separation of FFA from coffee extracts. Using isocratic elution, it was observed that the C8 column retained FFA substantially longer with better separation from other peaks and a satisfactory peak purity. FFA is a hydrophilic molecule that eluted faster by higher percentage of organic modifiers, thus the initial mobile phase composition was set to 0% of organic modifier. Later, a mild gradient slope using methanol was specifically developed to enhance peak shape and purity. Finally, different acidifiers (formic or acetic acid) at different percentages (0.0004–2%) were added to the aqueous part of the mobile phase to manipulate the pH with the aim of attaining a suitable retention time of the other three furan derivatives with good resolution. Acetic acid 0.1% was chosen as the aqueous part of the mobile phase for optimal separation of the four furan derivatives as seen in Figure 1. When the temperature of the column compartment was altered (17.5–35°C), shorter retention times were observed at temperatures above 30°C.

The final HPLC method took less than 15 min to completely separate the four furan derivatives with good peak shape and no observable co‐elution. Figure 1 shows a typical HPLC chromatogram of coffee analyzed using the developed method showing all four substances measured at the absorption maximum of the respective compound. Identification of the respective peaks was done by comparison of retention times and UV absorption spectra with those of authentic standards. The purity of the peaks was demonstrated using the integrated peak purity tool of the software. This peak purity tool detects impurities of less than 0.5% by comparing the spectra at the upslope, apex and downslope of the peaks of interest 20. Ground roasted coffee samples were analyzed directly after extraction without any further pre‐treatment.

### 3.2 Assessment of different extraction techniques

**Figure 2:**
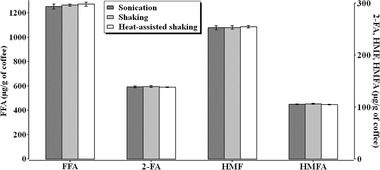
Concentrations (expressed as μg/g of coffee) of FFA, HMF, 2‐FA and HMFA that were extracted by the three different extraction techniques

Three different extraction techniques were applied to ground coffee to extract FFA, 2‐FA, HMF and HMFA. The same extraction solvent (water) and sample of ground coffee were used for the three techniques to allow a rational comparison of results. Figure 2 shows the results of the three extraction techniques. No significant differences were observed in the amounts of the four furan derivatives extracted by the three extraction techniques. This is due to the fact that the four furan derivatives of interest are highly water soluble and are easily extracted with water, independent of the extraction method used.

### 3.3 Assessment of the clean‐up of ground coffee

Three different extraction solvents were used to examine the clean‐up of FFA, 2‐FA, HMF and HMFA from ground coffee while extracting with heat‐assisted shaking for a fixed time, temperature and shaking conditions. Statistical treatment of the results shown in Table 1 revealed no significant differences between the extraction efficiencies of water and a 50% v/v water/methanol mixture regarding the four furan derivatives (Significance level α = 0.05). Both showed a minimum clean‐up in the first extraction round of 97.2% of the total amounts extracted of the four furan derivatives. Methanol presented no significant difference to the other two extraction solvents regarding the clean‐up of FFA from coffee (Significance level α = 0.05), however a significantly lower extraction efficiency of 2‐FA, HMF, and HMFA was observed (Significance level α = 0.05). The amounts extracted by methanol at the first extraction round was as low as 83.8% of the total amounts extracted of HMFA. These results point out that FFA is extracted efficiently using any of the tested solvents while the other examined furan derivatives were not extracted completely from ground roasted coffee with methanol.

**Table 1:** Results of two rounds of extraction applied to 50 mg of a commercial 100% Arabica coffee

	Water			50% v/v methanol in water			Methanol		
	A_1_	A_extra_	P (%)	A_1_	A_extra_	P (%)	A_1_	A_extra_	P (%)
FFA	64.0 (±0.71)	0.13 (±0.22)	0.20 (±0.35)	70.1 (±1.65)	0 (±0.0)	0 (±0.00)	80.3 (±5.10)	0.49 (±0.64)	0.62 (±0.80)
2‐FA	6.95 (±0.04)	0.05 (±0.02)	0.68 (±0.22)	7.68 (±0.27)	0.16 (±0.07)	2.09 (±0.92)	7.78 (±0.46)	1.22 (±0.21)	13.5 (±1.28)
HMF	12.8 (±0.12)	0.28 (±0.06)	2.17 (±0.45)	14.3 (±0.04)	0.26 (±0.07)	1.79 (±0.46)	13.3 (±0.80)	1.21 (±0.18)	8.30 (±0.69)
HMFA	5.26 (±0.02)	0.10 (±0.03)	1.81 (±0.58)	5.96 (±0.02)	0.16 (±0.01)	2.56 (±0.17)	5.07 (±0.28)	0.80 (±0.21)	13.5 (±2.35)

A_1_: amount (in μg) extracted by first extraction round corrected to 1 g of solvent. A_extra_: amount (in μg) extracted by second extraction round corrected to 1 g of solvent. P: percentage of the amount extracted by the second round to the total amount extracted. Values are given ± standard deviation (n = 3).

### 3.4 Validation of the analytical method

Table 2 shows all validation parameters obtained for the four furan derivatives in question. The acquired values for the correlation coefficient (*r* ^2^ ≥ 0.998), recovery (≥89.9%) and precision (intraday precision ≤ 4.2%, interday precision ≤ 4.5%) indicate that the developed method was linear, accurate and precise. The linearity was also tested by Mandel's test and a linear regression was accepted at a significance level of 99%. The obtained values of LOD and LOQ illustrate the sensitivity of the method. Although the detection wavelength for FFA (217 nm) is below the cut‐off of acetic acid, FFA could be detected with sufficient sensitivity due to the low acetic acid content (0.1%).

**Table 2:** Result values for the validation of the analytical method

	*r* ^2^	Slope	y intercept	Curve standard error (*n* = 15)	Intraday precision (RSD%)	Interday precision (RSD%)	LOD (μg/mL)	LOQ (μg/mL)	Recovery (%)
FFA	0.9982	11.07 (±0.34)	18.44 (±5.35)	3.70	0.68–4.21	0.71–3.33	0.76	2.55	91.00
2‐FA	0.9999	14.83 (±0.01)	−2.43 (±0.57)	1.24	0.14–1.11	0.28–4.50	0.12	0.41	97.57
HMF	0.9997	16.67 (±0.13)	−5.11 (±1.80)	2.19	0.12–0.97	0.81–3.30	0.11	0.35	89.94
HMFA	0.9998	11.64 (±0.08)	0.18 (±1.24)	1.09	0.43–1.04	0.22–3.54	0.23	0.78	99.57

The values of the slope and y intercept are given ± standard deviation (*n* = 3).

### 3.5 The analysis of coffee brews

The developed and validated method was applied successfully to the analysis of filter and espresso brews of a commercial 100% Arabica coffee prepared according to ISO 6668:2008. The analysis took place immediately after centrifugation of the brews. The resulting chromatograms were similar to that of the laboratory‐prepared extracts with no disturbing co‐elution from matrix peaks at the measured wavelengths, thus the method can be deemed as fast and appropriate for the routine analysis of these four furan derivatives. The levels of the four furan derivatives varied considerably in the prepared coffee brews between different furan derivatives and different brewing methods. FFA demonstrated the highest level of the analyzed derivatives at 66.6 ± 2.6 μg/mL filter brew and 71.7 ± 3.6 μg/mL espresso brew. The levels of HMF were at 28.5 ± 1.1 μg/mL filter brew and 29.2 ± 1.4 μg/mL espresso brew, which are noticeably lower than that of FFA. The detected levels of 2‐FA were at 8.0 ± 0.4 μg/mL filter brew and 8.4 ± 0.3 μg/mL espresso brew. HMFA levels were the lowest observed at 7.3 ± 0.4 μg/mL filter brew and 7.4 ± 0.4 μg/mL espresso brew.

## 4 CONCLUDING REMARKS

The extraction of four major furan derivatives from roasted coffee was examined. While similar extraction results were observed for different extraction techniques applied (water was used as the extraction solvent), significantly varying results ranging from 84–100% of the total extractable amounts were obtained with water, methanol, and water‐methanol mixtures as extraction solvents. Methanol was not suitable to completely extract 2‐FA, HMF, and HMFA from roasted coffee grounds. An improved HPLC‐DAD method using a C8 column was successfully established for the simultaneous determination of four major furan derivatives in roasted coffee. Fast separation and quantification of the volatile furan derivatives was carried out within a 15 min chromatographic run and with the application of minimum sample pre‐treatment. The results of the validation proved the linearity, accuracy, precision (intra and inter‐day), and sensitivity of the new method. Real coffee drip filter and espresso brews were analyzed with no disturbing co‐elution employing the developed HPLC method. The positive validation outcomes and the effective application of this method to actual coffee brews demonstrated the applicability of this HPLC method for the routine analysis of two potential genotoxic hydroxymethyl furan derivatives (FFA and HMF) alongside their carboxylic acid derivatives (2‐FA and HMFA, respectively) in order to simultaneously monitor their concentrations in roasted coffee, which is a widely consumed rich source of intake in regard to these furan derivatives.

## CONFLICT OF INTEREST

The authors have declared no conflict of interest.
